# Impact of teaching, learning, and assessment of medical law on cognitive, affective and psychomotor skills of medical students: a systematic review

**DOI:** 10.1186/s12909-023-04695-2

**Published:** 2023-09-26

**Authors:** Mahmoud Abbasi, Mandana Shirazi, Hojjat Torkmandi, Sanaz Homayoon, Mohammad Abdi

**Affiliations:** 1 Medical Ethics and Law Research Center, Shahid Beheshti University of Medical Sciences, Tehran, Iran; 2 Department of Medical Education, Faculty of Medicine, Tehran University of Medical Sciences, Tehran, Iran; 3 Department of Nursing, Zanjan University of Medical Sciences, Zanjan, Iran; 4 York university, Toronto, Canada

**Keywords:** Teach, Learn, Assessment, Law, Medical students, Knowledge, Attitudes, Performance

## Abstract

**Background:**

It is necessary to improve medical students’ legal cognitive, affective, and psychomotor skills to prevent further legal issues in the medical profession. Choosing the proper teaching and assessment methods is crucial in this matter. This study aimed to investigate the impact of teaching, learning, and assessment of medical law on the cognitive, affective, and psychomotor skills of medical students.

**Methods:**

A systematic review was conducted in PubMed, Embass, and Web of Science databases, and Google Scholar search engine using MECIR and PRISMA, AMEE Guide 94 for 1980 to 2022.12.30. Nineteen articles met the inclusion criteria. MERSQI checklist was used to assess the quality of the articles, and URSEC (theoretical underpinning of the development, resources required, setting, educational methods employed, and content) used to assess the risk of educational bias.

**Results:**

Internship courses called Medical Education Humanities and Society (MESH), clinical scenario design, seminars and small group discussions, web-based interactive training, legal training courses, PBL, and mind maps have been used to improve the medico-legal knowledge of medical students. MESH clerkship, simulation of a legal event, medico-legal advocacy program based on interdisciplinary education, group discussion, integration, and court-based learning used to improve student attitudes. Multidisciplinary training, small group discussions after the seminar, mock trial competition, and interdisciplinary education are used to teach psychomotor skills. All studies, except one on knowledge, reported positive effects of legal education on students’ knowledge, attitudes, and legal performance. Written assessments were used for cognitive and affective domains, while performance was assessed by OSCE, simulated court, and evaluation of patient referrals.

**Conclusion:**

There are few studies to examine the cognitive, affective, and legal psychomotor skills of medical students. The texts have not yet fully explored the high level of affective and psychomotor domains, which is evidence of a gap in this sector. Recognizing that medico-legal problems are prevented through proper education and assessment, it is recommended that this area be considered a research priority and that effective educational policies are adopted.

**Supplementary Information:**

The online version contains supplementary material available at 10.1186/s12909-023-04695-2.

## Background

Medicine has been generally accepted as one of the more respectable professions among people and has particular respect and social status [1]. People are hopeful to regain their health from a family physician, and people’s expectations in the medical profession are unique [2]. Now, if there are complications for the people to promote or regain their health while attending a physician due to shortcomings of the healthcare, it will cause disputes between physicians and patients [3]. Physicians and patients have rights and responsibilities towards one another that are addressed in medical ethics and laws [4]. Ethics offers advice to human beings in interactions with each other. However, by exploiting ethics as one of the sources of human rights, the law acts as a mediator to safeguard such actions [5].

Law is a set of rules that seeks to regulate behavior and practices using ethics, norms, jurisprudence, the constitution, common law, human rights statements, guidelines and expert opinions [6, 7]. The purpose of developing rules and regulations is to provide guidelines and regulate the interactions between healthcare providers and the public. This set of rules and regulations in medicine is presented under medical laws. Under these laws, physicians and patients are required to respect each other’s rights [4]. Applying these sets of rules and regulations in the clinic is considered one of the medical competencies.

The International Medical Education (IIME) sets out seven skills for physicians known as the ‘global minimum essential requirements’ (GMER) [8]: 1) Professional Values, Attitudes, Behavior, and Ethics, 2) Scientific Foundation of Medicine, 3) Clinical Skills, 4) Communication Skills, 5) Population Health and Health Systems, 6) Management of Information and 7) Critical Thinking and Research”.

The first skill is related to professional values, attitudes, and beliefs. Also, the roles of Health Advocacy and Professional in CANMEDS seven skills explicitly mention the legal skills of physicians [9]. Having such skills in physicians will reduce the legal problems of the health system. As mentioned in the texts of recent years, medical records have increased several times [10–12]. Increasing the number of cases and complaints from the medical staff will lower the trust element between physicians and patients [13] and lead to major time wasting for both patients and physicians due to frequent visits to medical clinics. It will also impose high costs on the healthcare system, medical staff, and patients [14]. However, these complaints are primarily preventable [15].

Educating physicians about legal issues and including these issues in students ‘curricula is the most important way to train physically qualified physicians to respect patients’ rights [16]. Legal concepts are presented in most of the world in the form of a subject called ethics and law, which are the main elements and the core fundamental of the medical curriculum [17]. These studies have mainly taught ethics, forensics, medical history, and medical jurisprudence in the curriculum, although the significant part is ethics and forensics, and the legal part has been neglected [18]. Given that the legal responsibilities of physicians are explicitly related to the science of law, attention to the education of their legal responsibilities is inevitable and should be considered in medical education [19]. Clarifying the civil and criminal responsibilities of physicians can create a healthy mental framework and motivate the legal identity of individuals to maintain the mutual rights of physicians and patients along with an understanding of ethics [20].

Unfortunately, the legal responsibilities of medical students are still not clearly defined in the Curriculum of Medical Ethics and Law, which is in effect in most countries around the globe [21]. The main goal of participatory law and medicine programs is primarily to ensure the implementation of laws affecting health, especially among vulnerable populations (pregnant mothers, the elderly, children, infants, the mentally ill, etc.) [22]. Teaching legal concepts in medical education programs can improve the community’s well-being, especially the vulnerable population, and remove barriers that can provide adequate health care [23]. Despite the importance of this issue, physicians’ knowledge of legal issues is still very scarce [24–26]. Therefore, one of the physicians’ most critical professional challenges is facing patients’ legal issues [10–12]. For effective law education, physicians need to consider aspects of competency that include cognitive, affective, and psychomotor skills. For this purpose, we can use approaches, principles, and models of medical education that examine learning-teaching and promoting professional competencies in medical sciences [27]. Accordingly, medical education generally offers various models of education, including Lecture, Team base Learning, Problem-Based Learning, Role Play, and Simulation-based practice [28]. Nevertheless, which of these methods is more effective in teaching law to medical students and what effect it has on increasing their knowledge, attitude, and practice are in a state of ambiguity. This gap led the authors of this article to conduct research aimed to investigate the impact of teaching, learning, and assessment of medical law on the cognitive, affective, and psychomotor skills of medical students.

## Methods

**Table 1:** Review of three valid systematic review guidelines including Cochrane Intervention Reviews (MECIR), AMEE Guide 94 and PRISMA

MECIR	PRISMA	AMEE Guide 94
**Developing the protocol for the review** 1.1 Setting the research question(s) to inform the scope of the review 1.2 Setting eligibility criteria for including studies in the review 1.3 Selecting outcomes to be addressed for studies included in the review 1.4 Planning the review methods at protocol stage **Performing the review** 1.5 Searching for studies 1.6 Selecting studies to include in the review 1.7 Collecting data from included studies 1.8 Assessing risk of bias in included studies 1.9 Synthesizing the results of included studies 1.10 Assessing the certainty of evidence and summarizing the findings	Protocol and registration Eligibility criteria Information sources Search Study selection Data collection process Data items Risk of bias in individual studies Summary measures Synthesis of results Risk of bias across studies Additional analyses	Step 1: Inception of review Step 2: Scoping searches Step 3: Assembling the full review team Step 4: Creating the protocol(work-plan) Step 5: Formulating the review question Step 6: Planning the search Step 7: Performing the search and selecting studies Step 8: Extracting data from the studies Step 9: Synthesising and analysing the data Step 10: Discussing and concluding the review Step 11: How the review will be reported

This systematic study investigated the teaching, learning, and assessment of law in medical education. Cochrane Intervention Reviews (MECIR) [29], AMEE Guide 94 [30], and PRISMA [31], were systematically reviewed to design the study (Table 1). Considering the many commonalities of these three guidelines and because the current study was of an educational type, we will describe it in detail based on the eleven steps of AMEE Guide 94 [30].

### Step 1: inception of review

Reflecting on the past ten years in the literature, it shows that the medical litigations have been rapidly increasing in this decade, and it has caused many problems and difficulties in the health care system. [10, 16, 32]. Many cases are due to physicians’ ignorance of legal issues in medicine [33]. Incompetence in this area can be a warning sign for physicians when dealing with patients to pay more attention [34]. Therefore, medical law training programs are needed to explain this issue. However, what about the process of teaching and evaluating medical law for medical students? What changes have educational interventions caused in students’ cognitive, affective, and legal psychomotor skills? It is a question that caught our attention.

### Research question

What is the impact of law education on the cognitive, affective, and psychomotor skills of medical students?

### Step 2: scoping searches

In the next step, we first did a basic search on the Cochrane database of systematic reviews, previous BEME reviews, the Database of Abstracts of Reviews of Effectiveness, and other electronic databases, such as PubMed and the google scholar search engine.

The study found that only one study was done in 2011 by Preston-Shoot [35], and this is a significant gap in the medical sciences. This study includes a review of effective outcomes in teaching, learning, and assessing law in medical education. Moreover, the review included studies from 1985 to 2009. However, It did not examine the educational effect in domains of legal cognitive, affective, and psychomotor skills. Studies have also been heterogeneous. While in the present study, only the effects of medical law education were considered, and true experimental, quasi experimental, mixed methods, educational interventional report and expo facto studies were selected.

### Step 3: assembling the full review team

In the third phase of our study, a research team consists of M.Sh. (experts in medical education and quality studies), M.A. (medical educator and legal advisor), M.A. (Attorney and Member of the Law School), H.H. (Healthcare provide and English Language Editor) and H.T. (Faculty member of the University of Medical Sciences and lawyer). H.T. and M.A. were eager to read articles in this area. SPSS v 20 software was used to analyze statistical data. We also employed two biostatistics and librarianship specialists to consult statistics and librarianship.

### Step 4: creating the protocol(work-plan)

The systematic review was conducted based on a pre-designed protocol to identify any reported studies or comments on medical legal education for medical students. The work was evaluated according to the Preferred Reporting Items for Systematic Reviews and Meta-analyses (PRISMA) and AMEE Guide 94 [30, 31].

### Step 5: formulating the review question

We used the PICO strategy to formulate the research question.

PICO: Population (Medical Student), Intervention /exposure (Curriculum, Teaching, Learning, Syllabus, Assessment); Comparative interventions (Not necessary for this study), and Outcomes of interest (Knowledge, attitudes, Opinion, Performance, Competency, Skill, behavior).

### Step 6: planning the search

#### Search strategy

In this study, related texts from the PubMed, Embase, and Web of Science databases and the Google Scholar search engine were reviewed using keywords (medical law, law, medicine, student, teaching, learning, assessment) and equivalent similar texts By developing keywords in the PubMed MeSH terms, Thesaurus dictionary, and using articles in this field, reached the following search strategy, which changed according to different databases and Google Scholar search engines (**Appendix1**).

((“Medical Student” OR “Student, Medical” OR " Medical School Enrollment” OR “Medical resident” OR “Medical residents” OR “Medical residency” OR Resident* OR residency OR “Student Run Clinic”) AND (Educat* OR “Education, Medical” OR “Medical edu*” OR Syllabus OR Program OR instruct* OR Pedagog* OR Teach* OR Learn* OR Train* OR Workshop OR Curriculum OR Curricula OR Curricular OR Assess* OR Evaluat* AND “Medical Law” OR “Law, Medical” OR “Medical Jurisprudence” OR “Jurisprudence, Medical” OR Medico-legal OR Law OR Laws OR Rule OR Rules OR legislations OR legislation OR Jurisprudence OR Regulation OR regulations OR Legal OR Malpractice OR Maltreatment OR “Legal Aspects” OR “Aspects, Legal” OR “Legal Aspect” OR Right OR Rights)) AND (Knowledge OR Awareness OR Understanding OR Information OR Attitude OR Opinion OR Sentiment OR Perception OR Vision OR Performance OR “Academic Performances” OR “Academic Performance” OR “Performance, Academic” OR Competency OR Competence OR “Clinical Competency” OR “Competency, Clinical” OR Skill OR “Clinical Skill” OR “Skill, Clinical” OR behavior).

The study period from 1980 to 2022.12.30 was included in the searches. Using the “snowball” method, we conducted our search by manually searching the reference lists of all available articles for other eligible studies [36].

The search strategy yielded 18,506 articles, which were reduced to 11,703 after removing duplicates. Based on the title and abstract screening, 550 articles were selected for full-text review. Eighteen articles met the inclusion criteria after reading the full text. One additional article was identified from the reference list. Therefore, a total of nineteen articles were included in the final analysis.

### Inclusion and exclusion criteria

We personalized the inclusion and exclusion criteria of the studies in terms of Population, Intervention, Comparison, and Outcome (PICO). The selection was limited to articles published in English. Criteria for entering studies in this project include:

#### Population

We included undergraduate and postgraduate medical students who received any academic law education (see Appendix 1 for keywords). We excluded studies that focused on school education, non-medical students, other paramedical disciplines (such as veterinary, dental, pharmacy, nursing, midwifery, and faculty studies), and educational reports for non-professionals.

#### Interventions

We selected studies that reported an educational intervention on medical students using any of the following designs: True experimental, quasi-experimental, mixed methods, educational interventional report, and expo facto. We included all types of legal education for medical students. We excluded studies that involved medical ethics and forensic medicine, or only considered the legal aspect of combined training. We did not review papers presented at conferences, seminars, letters to the editor, and authors’ personal opinions in detail.

#### Outcome

We included studies that reported the results of skill enhancement using any of the keywords in Appendix 1. We included all the results of the educational intervention that showed an effect on students’ knowledge, attitude, and performance. We excluded studies that did not report the learning outcome quantitatively (descriptive, analytical) or did not report the outcome at all.

### Step 7: performing the search and selecting studies

**Fig. 1:**
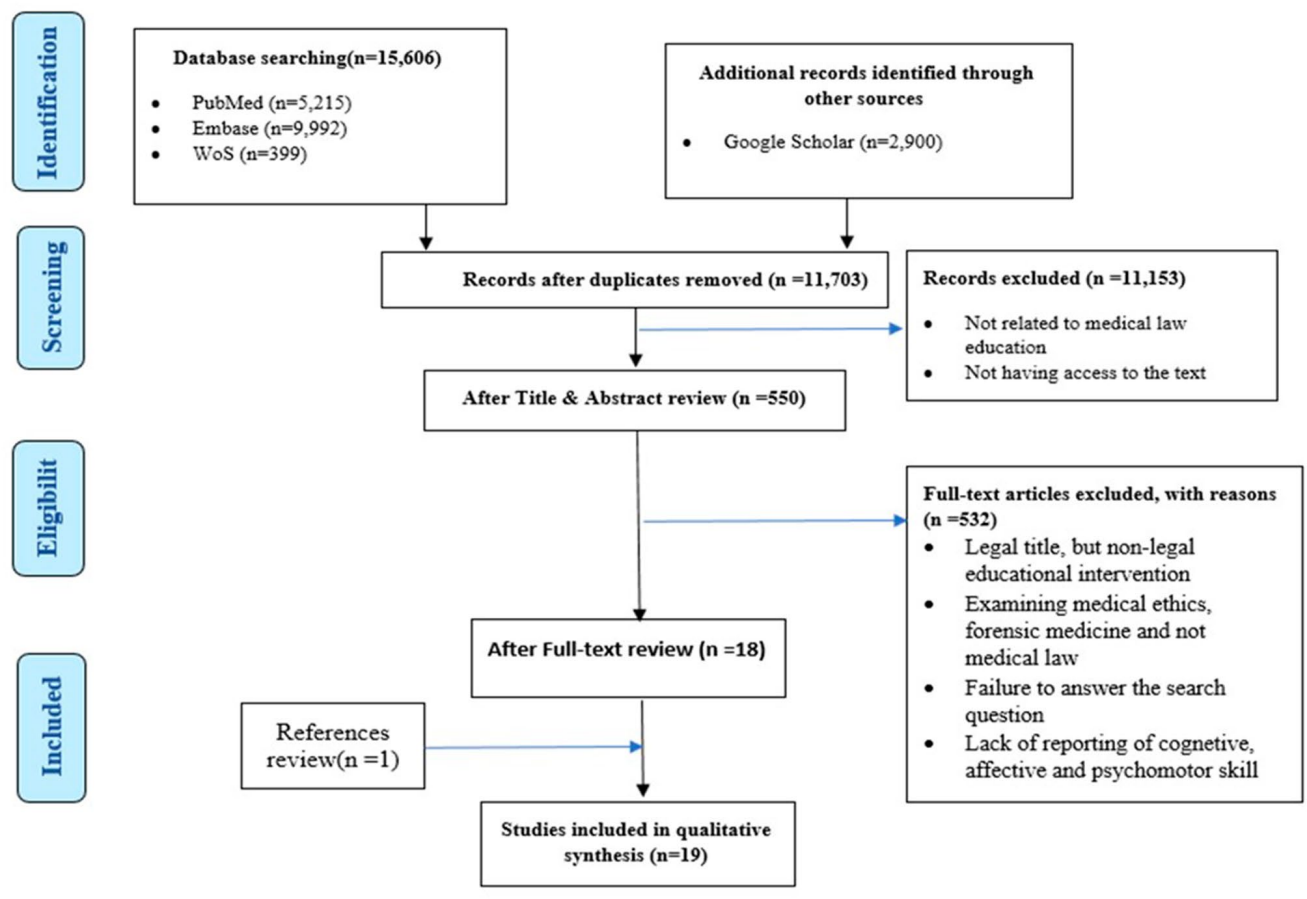
PIRISMA diagram for review studies

1. Article reviewers (H.T and M.A) initially looked only at titles, abstracts. They each shared the results of their studies separately. Kappa coefficient in the initial search of the title and abstract 0. 97. (600 H.T articles and 583 M.A) which reached 590 with a double review.
2. Out of 590 articles, 550 were available for full content review. Both researchers then read the full text of each article carefully) to make the decision on inclusion/exclusion. Out of 550 articles reviewed (58 H.T articles and 60 M.A articles), 18 cases were capable of detailed review. The agreement coefficient at this stage was 0.96. The reason for leaving out the articles was the lack of relevance to legal education, the inconsistency of the title with the text, the inconsistency with the purpose of this study, examining medical ethics, forensic medicine and not medical law, and the lack of reporting aspects of cognitive, affective and psychomotor skills. Also, some studies that had a legal title but the educational intervention was illegal were excluded from the study (Fig. 1; Table 2). We used the Medical Education Research Study Quality Instrument (MERSQI) to weight the studies [37].
3. An article was added with a review of references. Therefore, 19 articles were the final selection.

## Result

### Step 8: extracting data from the studies

**Table 2:** Studies in the field of medical law education to investigate cognitive, affective and legal Psychomotor skills of medical students

Author, Year	University, Country	Method	Population	Intervention	Outcome	MERSQI (1–18)
Le Blang, 1985 [38]	Southern Illinois, United States	Ex Post facto	Students in rotation of, Obstetrics and Gynecology psychiatry and pediatrics (n = 80).	Conduct a curriculum in internships called MESH Clerkship (Medical education Humanities and society) to teach medical law and evaluate students’ attitudes before and after the intervention with survey tools	Teaching medical law and regulations has a positive effect on medical students’ knowledge and attitudes about law and the legal system.	**11.5**
Goodnough, 1994 [39]	Case Western Reserve (5 Hospital), United States	Quasi	Third year general medicine students(n = 111).	1. an hour training session on erythrocyte transfusion standards 2. The 90-minute multidisciplinary workshop was with informed consent. 3. The effectiveness of the educational intervention was evaluated by the OSCE.	Effective for legal performance of the student.	**12**
Balcezak 1998 [40]	Yale-New Haven Hospital, CT, United States.	Quasi	All type of Residents (n = 100).	An educational website was designed on the subject of risk management and medical legal issues.	Effective way for increasing Knowledge.	**8.5**
J Goldie 2004 [41]	Glasgow, UK	Ex Post facto	A total of 101 students left the curriculum after year 3 to undertake an intercalated BSc, of whom 67 were from the cohort.	Prospective assessment of students in the curriculum developed to assess how to deal with a patient who is not satisfied with continuing treatment. The assessment was conducted through a written case and answers to four-choice questions and position descriptions.	Students’ legal-ethical attitudes increased after the implementation of the curriculum.	**10**
Kimberly E Liu,2005 [42]	Alberta, Canada	Quasi	Second-year medical students (n = 57).	surveys before and after an interdisciplinary panel session on ethics and legal issues in reproductive health.	Learners’ understanding of legal issues.	**11.5**
Bernice S Elge.2005 [43]	Geneva, Switzerland	Quasi	10 groups of medical students in years 4 and 5 and law students in years 1 and 2 were used (n = 311).	integral part of a teaching program about confidentiality and privacy rights. Case scenario assessment about breaching confidentiality before and after teaching.	Despite the theoretical training, participants did not fully understand the patient’s confidentiality obligations when they reached practical situations.	**6.5**
Nathaniel Ryan Schlicher, 2008 [44]	Simman laerdal medical Wappingers Falls, United States	Quasi	3-year accredited EM residency (n = 48).	Seven residents participated in a simulated program in which a nurse injected a patient with 1 gram of epinephrine and the patient suffered from anaphylactic shock. Attendees were asked to comment on the behaviors through a checklist.	The audience was very satisfied with this educational method. They also stated that this method affects their future behaviors.	**11.5**
Ellen Cohen, 2010 [45]	Boston, United States	Ex Post facto	MLPs educate residents (29 residency programs), health care providers (160 clinics and hospitals) and medical students (25 medical schools) (Resident of internal medicine, geriatrics, oncology, infectious disease, family medicine and pediatrics)	providing legal help to patients and working on policy advocacy Medico-legal partnership Data before and after evaluation of Legal Health training programs were collected from three hospitals.	Established MLPs have changed knowledge, attettide and performance.	**12.5**
Adele Evans, 2010 [46]	Wake Forest, United States	Quasi	graduating residents(n = 50).	2-day intensive medico-legal educational curriculum	improve physicians’ knowledge base regarding medico-legal interactions.	**10.5**
Sara Stafford, 2010 [47]	British Columbia, Canada	Quasi	111 residents in training with 43 post graduate year (PGY)-1, 33 PGY-2, and 35 PGY-3 residents.	medico-legal advocacy program Health Advocacy Questionnaire,	Increase students’ Advocacy attitude.	**9**
Malathi Srinivasan,2011 [48]	California Davis (UCD), California Los Angeles (UCLA), Washington, Seattle (UW), United States	Quasi	enrolled 80% of eligible residents at UCD (95/120), 75% at UCLA (154/205), and 38% at UW (30/78).	interactive, web-based curriculum on ethical, legal, and social implications in medical genetics for primary care residents. . Residents took five (of 10) cases and three (of five) tutorials that varied by specialty. We assessed changes in self-efficacy (primary outcome), knowledge, application, and viewpoints.	Baseline self-efficacy was improved.	**5.5**
DA Drukteinis, 2014 [49]	South Florida, Stetson, United States	Quasi (Mock trial competition (	emergency medicine residents (n = 10).	educational collaboration of Medicine and law. Residents dealt with a clinical case with a legal problem, then appeared in court with the help of a lawyer and testified.	helped them improve their communication and presentation skills in a litigious setting, expanded their understanding of the importance of proper documentation, illustrated problems caused by ineffective staff communication, and demonstrated the costly consequences of medical errors.	**8**
Kavita Shah Arora, 2014 [50]	Northwestern, United States	Ex Post facto	residents of obstetrics and gynecology (n = 39).	ethics educational interventions. The online survey was conducted with multiple-choice and open-ended questions for residents before and after the curriculum.	curriculum was demonstrated both improvement in confidence as well as knowledge of residents towards issues of reproductive ethics and law.	**13.5**
Shuh Shing Lee, 2016 [17]	Malaya, Malaysia	Quasi	first year medical students(n = 129).	A 4-week module was presented in the form of case studies. The program was in the form of group discussion and integration.	The evaluation was a written test. Based on students’ feedback, the introduction of medical law was found to be useful and hence an essential component to be integrated into the curriculum	**7**
Hui-Chin Chang, 2017 [51]	Chung Shan, Taiwan	Quasi	traditional approaches group (n = 68) in group I and II (n = 8) and III (n = 6) group were clinical PBL model teaching groups.	campus-based teaching group PBL model teaching groups with clinical case scenario	improve medical students general law knowledge.	**12.5**
Pettignano R [52]	Morehouse, United States	Interventional report	third-year medical student (n = 100)	medical students in the study, also attended by law students, focused on interprofessional collaboration to address client/patient SDH issues and health-harming legal needs.	increased likelihood to screen patients for SDH issues and an increased likelihood to refer patients to a legal resource.	**13.5**
Yanishevska, 2021 [53]	Sumy, Ukraine	Quasi	medical (n = 50) and legal students (n = 50).	interdisciplinary interactive teaching methods (Integration)	development of personal attitudes, future professional contacts, and practical skills.	**9**
S Siswati, 2021 [54]	Negeri Padang, Indonesia.	Quasi	Medical undergraduate students in control (n = 41) and experimental groups (n = 41).	using the ADDIE development method for determine effectiveness of PBL integrates with mind map model. students were divided into the experimental class and the control class. Pretest-Treatment-Posttest	Problem Based Learning by using mind mapping in the Health Law Ethics course was very effective improving learning outcomes.	**9.5**
Chen, WT., Fu, CP, 2022 [55]	National Defense Medical Center, Taiwan	mixed methods study	fourth-year medical students (n = 135).	Interdisciplinary court-based learning (CBL) component within the curriculum	Quantitative part: More than 60% of the students reported a comprehensive understanding in knowledge, attitudes, and skills in medical law from the CBL experience.	**8**

### The course of medical law education in litrature

During the review of full-text 550 studies published from 1985 to 2022 to select the final studies (19 studies), we noticed the evolution of medical law education, which we will describe. From 1966 to 1992, the published articles were mainly poorly structured. The turning point of this research was LeBlang in 1985, entitled “The impact of legal medicine education on medical students’ attitudes toward law.“ This examined the impact of curriculum planning (curriculum based on legal cases) required in medical law in the university medical school at Southern Illinois University was evaluated medical students’ knowledge and attitudes toward law and the legal system [38]. In this course, the main discussion of medical law education was based on the case report of educational interventions and personal views and without any particular methodology. These studies mainly introduced educational floods and recommendations for students and faculty members. For example, in an article, Oliver et al. (1986) addressed the importance of teaching law in medical sciences because, from their point of view, the legal responsibilities of the department. It is inalienability of the medical profession [56]. Measures needed to be taken in the medical curriculum to address these issues. Greaves et al. (1984) proposed recommendations for the inclusion of rights in the medical curriculum [57].

Since 1992, attention to various training in the form of cases and diseases (clinical = practical) has become more serious; for example, in the care of patients with AIDS, there are several legal challenges that in the form of more minor methodological studies to They were paid [58]. Later, controversial issues such as abortion became a concern for medical students [59]. The legal footprint has become much more prominent in a variety of medical staff tasks, such as medication [60], organ transplants [61, 62], and informed consent [63–66]. Qualifications advanced.

Another point is that due to the overlap of ethical and legal aspects in the medical curriculum, law education has continued with medical ethics since 1993. In other words, legal concepts were presented at the heart of the medical ethics curriculum. However, in the last decade, studies have primarily believed in separating ethical concepts from legal ones in medical education [18, 35, 67–72], and others were examined separately [73–77].

### The effect of law education on cognitive, affective and psychomotor skills of medical students (review of studies)

#### Cognitive

Out of 19 studies, ten assessed the knowledge and understanding of cognitive domain after teaching legal concepts to medical students. The first methodological study was conducted by LeBlang (1985), who evaluated the effect of a curriculum planning at the University of Southern Illinois University medical school. The curriculum was designed to create an internship program called MESH Clerkship (Medical education Humanities and society), which aimed to enhance the knowledge and attitude of medical students toward the law and the legal system. The study results showed an improvement in students’ knowledge and attitude [38].

Balcezak (1998) reported an increase in learners’ knowledge by designing a legal education website [40]. However, J Bernice S Elge (2005) et al., By developing a case-based scenario of privacy and privacy, acknowledged that theoretical instruction alone could not increase students’ understanding of practical situations [43]. Kimberly ELiu (2005) also monitored an interdisciplinary panel and reported that this course had improved learners’ understanding of legal issues, especially reproductive health [42]. In this regard, Cohen et al. (2010) used a Medico-legal partnership to change students’ legal knowledge, attitude, and performance [45].

Evans et al. (2010) designed a 4-day intensive medical and legal curriculum based on the Kollas model, which included seminars on legal topics. The students could discuss with their lawyer and judge in a small group at the end. This educational model improved learners’ knowledge in legal-medical interactions [46]. Srinivasan et al. (2011) reported that residents’ knowledge had increased by creating an interactive, web-based training program on the ethical, legal, and social implications of medical genetics for primary care residents [48]. Arora et al. (2014) conducted ethical-legal educational interventions on reproductive health. An online survey later revealed that the curriculum improves residents’ self-confidence and knowledge of ethics and reproductive rights issues [50]. ChinChang et al. (2017) compared two traditional methods, and PBL based on the scenario and the results showed that PBL had increased students’ legal knowledge [51]. Siswati et al. (2021) developed a PBL based on a mind map in medical ethics and law by designing the ADDIE model. After training, learners’ knowledge scores improved compared to before the training [54].

#### Affective

Out of 19 studies, eight articles were focused on affective domain. Part of LeBlang’s (1985) study was related to the field of students ‘attitudes, which also reported an increase in students’ attitudes [38]. Goldie (2004) examined students’ attitudes and encountered that they had improved through designing a clinical scenario for students (a patient who refuses to continue treatment) [41].

Schlicher (2008) marked the beginning of simulation programs for law education. In this simulated study, seven participants were to have an anaphylactic shock upon receiving 1gr of Epinephrine injection. Attendees were asked to comment on residents’ behaviors through a checklist. The results showed that the audience was satisfied with this educational method (score 4.63 out of 5). Also, they stated that this method affects their future behaviors [44].

Cohen et al. (2010) observed a Medico-legal partnership advocacy course, which was conducted together with physicians and lawyers, and reported that students’ attitudes increased after these programs. This study’s design was based on the interdisciplinary education method [45]. Stafford (2010) also developed a medico-legal advocacy program that effectively changed students’ attitudes. Students acknowledged that they would be more willing to participate in medico-legal advocacy programs in the future [47]. Lee et al. (2016) examined student feedback after a 4-week module in the case of law lectures in the form of group discussion and integration. The students stated that this legal course had prepared them mentally and physically when dealing with patients [17]. Yanishevska et al. (2021) reported that the program developed personal attitudes, future professional contacts, and practical skills by designing interdisciplinary, interactive teaching methods (Integration) and using a questionnaire [53]. Chen et al. (2022) used court-based learning to teach medical law in a mixed-method study. They reported the high satisfaction of students with this teaching method in the quantitative part of the research [55].

#### Psychomotor

Four of the 19 studies examined functional and behavioral skills. Goodnough (1994) reported an increase in students’ performance in the legal implementation of blood transfusions by conducting an educational intervention based on 1-hour training sessions on standards and legal issues and a 90-hour multidisciplinary workshop using the OSCE test [39]. In one part of the study, Cohen et al. (2010) reported that monitoring Medico-legal partnership advocacy courses based on interdisciplinary education increased patient referrals by residents [45]. Drukteinis et al. (2014) designed an educational collaboration of Medicine and law based on a mock trial competition in which residents first dealt with a clinical case with a legal problem, then appeared in court with the help of a lawyer and began to defend and testify themselves. This study showed that residents’ legal performance in testimony increased [49]. Pettignano et al. (2017) reported an increased probability of referring patients to a legal resource using interprofessional collaboration training [52].

### Educational impact

The educational impact is the assessment’s effect on the learner and the test taker [78]. Assessment’s first and most important purpose is to maximize students’ abilities by guiding and motivating them for further learning [79]. Unfortunately, this aspect has been very limited in studies. Nathaniel Ryan Schlicher et al. (2008) evaluated the effectiveness of training as an educational experience. They reported that the evaluation effectively changed participants’ behaviors and opinions about the negligence system [44]. Srinivasan et al. (2011) reported that the training program and assessment could increase self-learning in students. However, his conclusion was related to the whole program, and the trial was not specifically on the examination [48]. After holding a mock trial competition, Dainius A. Drukteinis et al. (2014) reported that residents noted various educational implications, including learning how to appear in court and testify appropriately [49]. Hui-Chin Chang (2017) et al. reported that the PBL course stimulated students’ study but did not mention the educational effect of the assessment [51]. In general, this part has been neglected in studies.

### Risk of bias in reporting educational interventions in included studies

The theoretical **u**nderpinning of the development, the **r**esources required, the **s**etting, the **e**ducational methods employed, and the **c**ontent.

Selected articles were evaluated for bias in educational interventions using a bias risk criterion developed by Gordon et al. (2021), quoted by Guckian et al. [80]. These papers include five potential bias sources (URSEC) that include the theoretical **u**nderpinning of the development, the **r**esources required, the **s**etting, the **e**ducational methods employed, and the **c**ontent.

Each resource is considered low risk by a three-point scale that includes articles that provide adequate explanations. Those that provide details but are inadequate are rated as uncertain bias risk, and those that provide no details are rated as risk above bias [81, 82].

Based on the five-source bias scale extracted from BEME in general, the risk of bias in reporting educational interventions was reasonable (Table 3). In none of the sources was the High-Risk bias above 40%. Resource bias was the most common, as studies did not clearly explain the costs, time, and resources required for the development. However, the least biased source was the educational setting, which was often explicitly explained.

**Table 3:** Risk of bias in studies (N = 19)

Source of Bias Level of Risk	Low Risk N(%)	Medium Risk N(%)	High Risk N(%)
Underpinning	9 (47.36)	4 (21.05)	6(31.59)
Resources required	5 (26.33)	6(31.57)	8(42.10)
Setting	11(57.89)	5(26.33)	3(15.78)
Educational methods	10 (52.65)	3(15.78)	6(31.57)
Content	8(42.10)	6(31.57)	5(26.33)

### Step 9: synthesising and analysing the data

The quality of the studies was assessed using MERSQI, and the mean (standard deviation) score was 9.92(2.46) (Table 4). Out of 19 studies, 7 (36.8%) used a single group posttest only design, and none used a RCT design. Most studies (73.7%) recruited participants from one institution. Fourteen studies (73.7%) had more than 75 subjects in the intervention and control groups. Only 4 (21.1%) studies collected data through observation, while the rest used questionnaires. The instruments used were often poorly described in terms of structure 10 (52.6%), content 13 (38.4%), and validity 10 (52.6%). The analysis was descriptive or beyond descriptive 8 (42.1%). Most studies measured knowledge outcomes (42.1%), and only two studies examined patient/healthcare outcomes.

**Table 4:** Medical Education Research Quality Instrument for quantitative studies

Domain	MERSQI Item	Score	N(%)	M (SD) of Score	M (SD) of Domain
Study design	Single group cross-sectional or single group posttest only	1	7(36.8)	0.36(0.49)	1.42(0.38)
	Single group pretest & posttest	1.5	8(42.1)	0.63(0.76)	
	Nonrandomized, 2 groups	2	4(21.1)	0.42(0.83)	
	Randomized controlled trial	3	0(0)	0(0)	
Sampling	*Institutions studied*:				2.07(0.50)
	1	0.5	14(73.7)	0.36(0.22)	
	2	1	2(10.5)	0.05(0.22)	
	3	1.5	4(21.1)	0.31(0.62)	
	*Response rate, %*:				
	Not applicable				
	< 50 or not reported	0.5	1(5.3)	0.02(0.11)	
	50-74	1	4(21.1)	0.21(0.41)	
	> 75	1.5	14(73.7)	1.10(0.67)	
Type of data	Assessment by participants	1	15(78.9)	0.78 (0.41)	1.42(0.83)
	Objective measurement	3	4(21.1)	0.63 (1.2)	
Validity of evaluation instrument	*Internal structure*:				1.26(1.19)
	Not applicable				
	Not reported	0	10(52.6)	0(0)	
	Reported	1	9(47.4)	0.47(0.51)	
	*Content*:				
	Not applicable				
	Not reported	0	13(68.4)	0(0)	
	Reported	1	6(31.6)	0.31(0.47)	
	*Relationships to other variables*:				
	Not applicable				
	Not reported	0	10(52.6)	0(0)	
	Reported	1	9(47.4)	0.47(0.51)	
Data analysis	*Appropriateness of analysis*:				2.21(0.78)
	Inappropriate for study design or type of data	0	5(26.3)	0(0)	
	Appropriate for study design, type of data	1	18(94.7)	0.94(0.22)	
	*Complexity of analysis*:				
	Descriptive analysis only	1	8(42.1)	0.42(0.50)	
	Beyond descriptive analysis	2	8(42.1)	0.84(1.01)	
Outcomes	Satisfaction, attitudes, perceptions, opinions, general facts	1	7(36.8)	0.36 (0.49)	1.52(0.61)
	Knowledge, skills	1.5	8(42.1)	0.63 (0.76)	
	Behaviors	2	2(10.5)	0.21 (0.63)	
	Patient/health care outcome	3	2(10.5)	0.31 (0.94)	
Total possible score*		19	19(100)		9.92(2.46)

*Scores range from 5 to 18. Adapted from Reed DA et al. Association between funding and quality of published medical education research. JAMA 2007; 298:1002–9

## Discussion

### Step 10: discussing and concluding the review and step 11: how the review will be reported

#### Cognitive

The results of the present study showed that, in order to improve the legal cognitive skills of medical students from internships called MESH (one case), clinical scenario design (two cases), seminar and small group discussion (one case), interactive and non-interactive web-based education (two cases), law training course (one case), PBL (two cases) and mind map (one case) were used.

Knowledge education in medical education can be divided into two categories teacher-centered and student-centered. In the teacher-centered category, passive learning methods are mostly used, and the role of the learner is minimal, as the teacher delivers the content as a monologue [83]. These include traditional lectures, demonstration methods, monologue team teaching, journal clubs without discussion, monologues, and individual-only reading [28, 84]. All of this enhances knowledge at the level of remember and understanding of Bloom’s taxonomy. On the other side of the spectrum are active learning methods such as PBL, Gamification, TBL, project base learning, scenario base learning, active demonstration, group discussion and other dialogue-based methods that cause involvement and inclusive participation in the teaching-learning process [85–87]. Some modern methods for enhancing knowledge have been developed, such as computer training, mentoring training, and e-mentoring program [88]. These methods are examples of active learning, which can promote learners’ knowledge to the higher levels of Bloom’s taxonomy, such as application, analysis, synthesis, and evaluation [89].

Lectures are the most common form of medical law education [35]. However, Elger et al. (2005) [43] and Vallée et al. (2020) [90] argued that lectures alone do not foster high levels of legal cognitive skills. This issue is controversial, as some studies, such as Alaagib et al. (2019) [91], AlQhtani et al. (2021) [92], and Golden et al.(1989) [93] reported that the lecture method is cost-effective, appropriate, common, and effective in teaching medical science fields. They claimed that the lecture method can lead to high-level learning in students if the techniques are used correctly. On the other hand, other studies have recommended the use of Scenario Base Learning and PBL methods to achieve the top of the knowledge pyramid [94]. They suggested that using the patient’s language in the clinical scenario can create a sense of empathy and problem-solving for the learner [23, 95]. Elge et al.(2005) also used clinical scenarios for student education [43]. Besides clinical scenarios, clinical methods (workplace-based) and direct communication with the patient can also be used to teach law to medical students. For instance, Le Blang (1985) used psychomotor methods (designing a MESH internship) to improve legal knowledge [38]. However, these studies did not assess the higher levels of cognitive skills in Bloom’s taxonomy (reasoning, evaluation, and synthesis). Therefore, future studies should pay more attention to this category.

In some exceptional circumstances (such as COVID-19 or the limitations of training centers), in-person clinical training is not possible. Therefore, work-place based education is not possible throughout the course, so using E-learning methods can be helpful if there are such limitations. In this regard, Srinivasan et al. (2011) designed a web-based interactive training program to teach legal concepts [48]. In this study, the interaction details were not mentioned, but in the texts, interactions in the virtual environment were mentioned, which can be used. Interactions in the virtual environment can be student-teacher interaction, student-content interaction, and student-student interaction [95]. In the virtual environment, engaging the student can provide high levels of knowledge for him [96]. Even with the advancement of technology and the advent of Tele-nursing and Telemedicine, student-patient interaction can be shaped virtually [97]. However, the legal aspects of virtual care and education in this area are a big gap for medical groups and require the intervention of educational planners. In the studies studied, various methods were used to increase students’ legal knowledge, but students’ level of involvement and activity was not fully reported. In other words, no report on their activity level is available.

All of the studies evaluated knowledge using written methods. Written tests are suitable for assessed knowledge alone [98]. However, to measure the higher levels of Bloom’s taxonomy, it is recommended that these assessments should not rely on a questionnaire, as it may not reflect the student’s actual situation [43]. Instead, individual projects based on medical law scenarios and their solutions by the student can be used for this purpose. This way, it is possible to evaluate the overall cognitive ability and provide a more meaningful learning effect for the student. These methods mainly promote learning by creating reflection [99].

#### Affective

Internships called MESH (one case), simulation of a legal event (one case), medico-legal advocacy program based on Inter-disciplinary education (two cases), group discussion and interdisciplinary, interactive teaching methods (Integration) (one case) were used to improve the medical students’ attitude regarding the laws.

The attitudinal learning domain is based on values, beliefs, culture, customs, and traditions [100]. Teaching attitude in medical sciences is mainly student-centred. In order to create attitudinal capacities in individuals, they must be involved in the educational process [101]. For this purpose, discussion methods, research, film, question and answer, role model, role play, mentoring, and community-based medical education (CBME) are used. These methods are based on interaction with peers, community, patients, co-workers, and others [100, 102]. In the studies we examined, the methods were mainly in the form of Inter-disciplinary and Integration.

Each person’s attitude is formed through experiencing different situations in life, in which a person develops personal views of life and themselves and how they react to them [103]. Thus, for effective education, the learner should experience a particular situation (real-simulated). For example, the study of Schlicher (2008) and colleagues created a simulated scenario to examine the audience’s attitude toward legal issues [44]. Cohen et al. (2010) also used Medico-legal partnership advocacy courses to train and examine legal attitudes [45]. The literature review showed that the legal attitude of students has increased by short term medical law training courses. But there is no consensus on this issue in other studies, Mata et al. (2021) [104] and Laal et al. (2021) [105] reported that attitude improvement requires continuous and long-term courses and is not possible with short training sessions. However, the literature review shows that attitudes are a learned tendency to value things in a certain way and that they can change based on various factors such as experience, social factors, and cognitive dissonance [106, 107]. It explains that cognitive dissonance is a psychological mechanism that occurs when there is an inconsistency between one’s attitudes and behavior and that people tend to reduce this dissonance by changing their attitudes according to their behavior. It suggests that short-term training can lead to attitude changes by inducing cognitive dissonance in participants, who may then adjust their attitudes to justify their participation in the course [108]. Also, short-term training courses may cause attitude change by influencing self-perception, as the participants may infer their attitudes from their behaviors during the education [109].

Most studies in medical law education have focused on the lower levels of the emotional pyramid, while neglecting the upper levels. Rogers (2017) cited Krathwohl’s five levels for the emotional domain, which are receiving, responding, valuing, organizing, and characterizing [110]. Future studies should pay more attention to this issue in medical law education. However, evaluating behavioral outcomes and objectives in the emotional domain is challenging, as it involves measuring the level of attention, attitude and depth of values acquired. This is not a one-time task, but requires continuous care, observation and extensive experience in many cases [111].

Emotional evaluations were also limited to written methods and questionnaires, which do not seem suitable for achieving a high level of professional commitment, respect for patients’ rights, adherence to professional values, and other issues in the field. Instead, alternative study methods such as observational evaluation, interviews, 360 degrees, portfolio, online system, video base, peer assessment in the workplace, or a simulated way can be used [112–116].

#### Psychomotor

In the reported studies, to improve the functional skills of law in medical students, they used multidisciplinary training sessions (two case), small group discussions after the seminar (one case), and mock trial competition (one case).

Other methods such as simulation-based training, clinical internship, real-world scenario-based training, morning, mentor and mentee, longitudinal integrated clerkships (LICs), bedside teaching, shift reports, and ground rounds to teach functional skills in medical sciences are available that integrate the psycho-motor, and students would consider them to be more practical [117–119]. However, implementing these methods requires the participation of other disciplines, which are primarily present outside the hospital setting. Teaching complex clinical procedures such as intubation only requires the presence of a physician and nurse. In contrast, for real law education in Medicine, disciplines such as lawyers, judges, and jurists are also needed [46]. Medical law education requires a broader interdisciplinary team. With this in mind, Drukteinis et al. (2014) designed an educational collaboration of medicine and law based on a mock trial competition and used an interdisciplinary team to teach legal concepts [49]. We can increase students’ legal capacity and protect patients’ rights by creating simulated situations in the workplace. We can even use emerging training such as virtual reality (VR), augmented reality (AR), gamification, computer base-training, or simulated trials and participatory camps for law and medical students. Of course, studies have shown that using these methods is costly, time-consuming, and requires training for teachers and students [120, 121]. However, with appropriate educational planning and management, any teaching method can be applied according to the needs of the learners and the facilities of the institution.

The OSCE test (two cases) and the simulated trial (one case) were used for the evaluation. Performance tests should be used to assess legal performance. For this purpose, 360-degree tests, OSCE, laboratory-based tests, long case, short case, DOPS and mini CEX can be used. In addition, student performance can be assessed in an actual or simulated trial.

## Conclusion

Law education positively affects students’ cognitive, affective, and psychomotor skills. However, there has been limited publication in this area; for instance, high levels of affective and psychomotor domains have not been studied. Choosing proper education and assessment methods can increase medical students’ cognitive, affective, and legal psychomotor skills in order to have more competent physicians in terms of respecting patient rights, reducing legal problems and saving time and money for physicians and patients. Given the many educational gaps, it is recommended to conduct more studies in this area and to take current achievements into account by health education policymakers.

### Electronic supplementary material

Below is the link to the electronic supplementary material.

Supplementary Material 1

## Data Availability

The dataset supporting the conclusions of this article is included within this article. The search strategy in PubMed, Embase, Web of Science and Google Scholar is available in the supplementary file titled as Appendix 1.
