# Supplementary material for: Impact of teaching, learning, and assessment of medical law on cognitive, affective and psychomotor skills of medical students: a systematic review
Source: BMC Med Educ. 2023 Sep 26;23:703. doi: 10.1186/s12909-023-04695-2 (PMC10523676; doi:10.1186/s12909-023-04695-2)
Supplement: Supplementary file 1 — Supplementary Material 1 [file 12909_2023_4695_MOESM1_ESM.docx]

**Appendix 1 : The search strategy used in each database**

|  | Database | Search Strategy |
| --- | --- | --- |
| 1 | Pubmed  5,215 | (("Medical Student"[Title/Abstract] OR "Student, Medical"[Title/Abstract] OR " Medical School Enrollment"[Title/Abstract] OR "Medical resident"[Title/Abstract] OR "Medical residents"[Title/Abstract] OR "Medical residency"[Title/Abstract] OR Resident*[Title/Abstract] OR residency[Title/Abstract] OR "Student Run Clinic"[Title/Abstract]) AND (Educat*[Title/Abstract] OR "Education, Medical"[Title/Abstract] OR "Medical edu*"[Title/Abstract] OR Syllabus[Title/Abstract] OR Program[Title/Abstract] OR instruct*[Title/Abstract] OR Pedagog*[Title/Abstract] OR Teach*[Title/Abstract] OR Learn*[Title/Abstract] OR Train*[Title/Abstract] OR Workshop[Title/Abstract] OR Curriculum[Title/Abstract] OR Curricula[Title/Abstract] OR Curricular[Title/Abstract] OR Assess*[Title/Abstract] OR Evaluat*[Title/Abstract] AND "Medical Law"[Title/Abstract] OR "Law, Medical"[Title/Abstract] OR "Medical Jurisprudence"[Title/Abstract] OR "Jurisprudence, Medical"[Title/Abstract] OR Medico-legal[Title/Abstract] OR Law[Title/Abstract] OR Laws[Title/Abstract] OR Rule[Title/Abstract] OR Rules[Title/Abstract] OR legislations[Title/Abstract] OR legislation[Title/Abstract] OR Jurisprudence[Title/Abstract] OR Regulation[Title/Abstract] OR regulations[Title/Abstract] OR Legal[Title/Abstract] OR Malpractice[Title/Abstract] OR Maltreatment[Title/Abstract] OR "Legal Aspects"[Title/Abstract] OR "Aspects, Legal"[Title/Abstract] OR "Legal Aspect"[Title/Abstract] OR Right[Title/Abstract] OR Rights[Title/Abstract])) AND (Knowledge[Title/Abstract] OR Awareness[Title/Abstract] OR Understanding[Title/Abstract] OR Information[Title/Abstract] OR Attitude[Title/Abstract] OR Opinion[Title/Abstract] OR Sentiment[Title/Abstract] OR Perception[Title/Abstract] OR Vision[Title/Abstract] OR Performance[Title/Abstract] OR "Academic Performances"[Title/Abstract] OR "Academic Performance"[Title/Abstract] OR "Performance, Academic"[Title/Abstract] OR Competency[Title/Abstract] OR Competence[Title/Abstract] OR "Clinical Competency"[Title/Abstract] OR "Competency, Clinical"[Title/Abstract] OR Skill[Title/Abstract] OR "Clinical Skill"[Title/Abstract] OR "Skill, Clinical"[Title/Abstract] OR behavior[Title/Abstract]) |
| 3 | Web of Science  339  results | (((ALL=("Medical Student" OR "Student, Medical" OR " Medical School Enrollment" OR "Enrollment, Medical School" OR Medical School Enrollments OR "Medical resident" OR "medical residents" OR "medical residency" )) AND ALL=(Education OR "Education, Medical" OR "Medical edu*" OR Syllabus OR Program OR instruct* OR Pedagog* OR Teach* OR Learn* OR Train* OR Workshop OR curriculum OR curricula OR curricular OR Assess* OR Evaluat* )) AND ALL=("Medical Law" OR "Law, Medical" OR Medico-legal OR Law OR Laws OR rule OR rules OR Legal OR Malpractice OR Maltreatment )) AND ALL=(Knowledge OR attitudes OR Opinion OR Performance OR "Academic Performances" OR "Performance, Academic" OR Competency OR Competence OR "Clinical Competency" OR "Competency, Clinical" OR Skill OR "Clinical Skill" OR "Skill, Clinical" OR behavior) |
| 4 | Google Scholar  2900 | allintitle: (((Medical) AND ((education OR instruction OR Pedagogy OR Teach OR Learn OR Train OR curriculum)) AND (Law OR Medico-legal OR rule OR legislation OR Legal OR Malpractice))) |
| 5 | Embase  9992 | ('medical student':ab,ti OR 'student, medical':ab,ti OR 'medical school enrollment':ab,ti OR 'medical resident':ab,ti OR 'medical residents':ab,ti OR 'medical residency':ab,ti OR resident*:ab,ti OR residency:ab,ti OR 'student run clinic':ab,ti) AND ((educat*:ab,ti OR 'education, medical':ab,ti OR 'medical edu*':ab,ti OR syllabus:ab,ti OR program:ab,ti OR instruct*:ab,ti OR pedagog*:ab,ti OR teach*:ab,ti OR learn*:ab,ti OR train*:ab,ti OR workshop:ab,ti OR curriculum:ab,ti OR curricula:ab,ti OR curricular:ab,ti OR assess*:ab,ti OR evaluat*:ab,ti) AND 'medical law':ab,ti OR 'law, medical':ab,ti OR 'medical jurisprudence':ab,ti OR 'jurisprudence, medical':ab,ti OR 'medico legal':ab,ti OR law:ab,ti OR laws:ab,ti OR rule:ab,ti OR rules:ab,ti OR legislations:ab,ti OR legislation:ab,ti OR jurisprudence:ab,ti OR regulation:ab,ti OR regulations:ab,ti OR legal:ab,ti OR malpractice:ab,ti OR maltreatment:ab,ti OR 'legal aspects':ab,ti OR 'aspects, legal':ab,ti OR 'legal aspect':ab,ti OR right:ab,ti OR rights:ab,ti) AND (knowledge:ab,ti OR awareness:ab,ti OR understanding:ab,ti OR information:ab,ti OR attitude:ab,ti OR opinion:ab,ti OR sentiment:ab,ti OR perception:ab,ti OR vision:ab,ti OR performance:ab,ti OR 'academic performances':ab,ti OR 'academic performance':ab,ti OR 'performance, academic':ab,ti OR competency:ab,ti OR competence:ab,ti OR 'clinical competency':ab,ti OR 'competency, clinical':ab,ti OR skill:ab,ti OR 'clinical skill':ab,ti OR 'skill, clinical':ab,ti OR behavior:ab,ti) AND [<1966-2022]/py |
